# Training of midwives in advanced obstetrics in Liberia

**DOI:** 10.2471/BLT.15.160473

**Published:** 2016-05-02

**Authors:** Obed Dolo, Alice Clack, Hannah Gibson, Naomi Lewis, David P Southall

**Affiliations:** a CB Dunbar Maternity Hospital, Gbanga, Liberia.; b Maternal and Childhealth Advocacy International, Brikama Major Health Centre, Brikama, Gambia.; c Maternal and Childhealth Advocacy International, 1 Columba Court, Laide, IV22 2NL, Scotland.

## Abstract

**Problem:**

The shortage of doctors in Liberia limits the provision of comprehensive emergency obstetric and neonatal care.

**Approach:**

In a pilot project, two midwives were trained in advanced obstetric procedures and in the team approach to the in-hospital provision of advanced maternity care. The training took two years and was led by a Liberian consultant obstetrician with support from international experts.

**Local setting:**

The training took place in CB Dunbar Maternity Hospital. This rural hospital deals with approximately 2000 deliveries annually, many of which present complications. In February 2015 there were just 117 doctors available in Liberia.

**Relevant changes:**

In the first 18 months of training, the trainees were involved with 236 caesarean sections, 35 manual evacuations of products of conception, 25 manual removals of placentas, 21 vaginal breech deliveries, 14 vacuum deliveries, four repairs of ruptured uteri, the management of four cases of shoulder dystocia, three hysterectomies, two laparotomies for ruptured ectopic pregnancies and numerous obstetric ultrasound examinations. The trainees also managed 41 cases of eclampsia or severe pre-eclampsia, 25 of major postpartum haemorrhage and 21 of shock. Although, initially they only assisted senior doctors, the trainees subsequently progressed from direct to indirect supervision and then to independent management.

**Lessons learnt:**

To compensate for a shortage of doctors able to undertake comprehensive emergency obstetric and neonatal care, experienced midwives can be taught to undertake advanced obstetric care and procedures. Their team work with doctors can be particularly valuable in rural hospitals in resource-poor countries.

## Résumé

**Problème:**

Au Libéria, la pénurie de médecins limite la fourniture de soins complets en obstétrique et néonatologie d'urgence.

**Approche:**

Dans le cadre d'un projet pilote, deux sages-femmes ont été formées aux procédures obstétricales avancées ainsi qu'à l'approche d'équipe à adopter pour pratiquer des soins avancés de maternité en milieu hospitalier. Cette formation a duré deux ans. Elle a été dirigée par un consultant obstétricien libérien, avec le soutien d'experts internationaux.

**Environnement local:**

La formation a eu lieu au CB Dunbar Maternity Hospital. Cet hôpital rural gère environ 2 000 accouchements par an, dont beaucoup présentent des complications obstétricales. En février 2015, seuls 117 médecins étaient disponibles au Libéria.

**Changements significatifs:**

Au cours des 18 premiers mois de la formation, ces sages-femmes ont participé à 236 accouchements par césarienne, 35 évacuations manuelles des produits de conception, 25 extractions manuelles du placenta, 21 accouchements par voie basse avec présentation par le siège, 14 accouchements par ventouse obstétricale, 4 réparations de ruptures utérines ainsi qu'à la prise en charge de 4 cas de dystocie des épaules, 3 hystérectomies, 2 laparotomies pour des grossesses extra-utérines rompues et à de nombreuses échographies obstétricales. Les bénéficiaires de la formation ont également géré 41 cas d'éclampsies ou de pré-éclampsies graves, 25 cas d'hémorragies du post-partum importantes et 21 cas de chocs. Même si, initialement, les bénéficiaires de la formation n'ont fait qu'assister des médecins expérimentés, par la suite, elles sont progressivement passées d'une supervision directe à une supervision indirecte jusqu'à intervenir de façon indépendante.

**Leçons tirées:**

Pour compenser une pénurie de médecins capables de pratiquer des soins complets en obstétrique et néonatologie d'urgence, il est possible de former des sages-femmes expérimentées aux procédures et soins obstétricaux avancés. Leur intervention en équipe auprès des médecins peut s'avérer particulièrement précieuse dans les hôpitaux ruraux des pays à faibles ressources.

## Resumen

**Situación:**

La escasez de médicos en Liberia reduce la disponibilidad de atención obstétrica y neonatal de emergencia completa.

**Enfoque:**

En un proyecto piloto, dos pateras recibieron formación en procesos obstétricos avanzados y en el enfoque de equipo sobre la atención materna avanzada hospitalaria. La formación duró dos años y estuvo dirigida por un asesor obstetra liberiano, con apoyo de expertos internacionales.

**Marco regional:**

La formación se realizó en el CB Dunbar Maternity Hospital. Este hospital rural se ocupa de alrededor de 2 000 partos al año, muchos de los cuales presentan complicaciones. En febrero de 2015, únicamente había 117 médicos disponibles en Liberia.

**Cambios importantes:**

Durante los primeros 18 meses de formación, las alumnas participaron en 236 cesáreas, 35 evacuaciones manuales de productos de la concepción, 25 extracciones manuales de placentas, 21 partos de nalgas por vía vaginal, 14 partos de extracción por vacío, 4 reparaciones de desgarros uterinos, la gestión de 4 casos de distocia del hombro, 3 histerectomías, 2 laparotomías para rupturas de embarazos ectópicos y numerosas ecografías obstétricas. Las alumnas también se enfrentaron a 41 casos de eclampsia o preeclampsia grave, 25 de hemorragias posparto intensas y 21 de shock. A pesar de que al principio únicamente asistían a médicos, las alumnas fueron progresando de supervisión directa a indirecta y llegaron a realizar una gestión independiente.

**Lecciones aprendidas:**

Para compensar la escasez de médicos disponibles para atención obstétrica y neonatal de emergencia completa, las parteras con experiencia pueden aprender a realizar procedimientos y medidas de atención obstétrica avanzada. Trabajan en equipo con médicos, lo que puede resultar especialmente valioso en hospitales rurales de países con pocos recursos.

## ملخص

**المشكلة:**

يؤدي نقص الأطباء في ليبيريا إلى تقييد فرص توفير الرعاية التوليدية الطارئة الشاملة ورعاية الأطفال الولدان.

**الأسلوب:**

في أحد المشاريع الارتيادية، تم تدريب اثنتين من القابلات لمعرفة إجراءات التوليد المتقدمة وأسلوب الفريق المتبع في توفير الرعاية الأمومية المتقدمة في المستشفى. استغرق التدريب سنتين تم تحت قيادة تحت قيادة طبيب توليد استشاري ليبيري مع توفير الدعم له من جانب الخبراء الدوليين.

**المواقع المحلية:**

انعقد التدريب في مستشفى التوليد CB Dunbar. وتتعامل هذه المستشفى الريفية مع ما يقرب من 2000 حالة ولادة سنويًا تواجه العديد منها بعض المضاعفات. وفي فبراير/شباط من عام 2015، توفر فقط ما يصل إلى 117 طبيبًا في ليبيريا.

**التغيّرات ذات الصلة:**

في أول 18 شهرًا من التدريب، شارك المتدربون في إجراء 236 حالة جراحة قيصرية، و35 عملية إخراج يدوية لمنتجات التلقيح، و25 عملية إزالة يدوية للمشيمة، و21 حالة ولادة مقعدية مهبلية، و14 حالة ولادة باستخدام أجهزة شفط الجنين، وأربعة إصلاحات لتمزق الرحم، وإدارة أربعة حالات لعسر الولادة من اتجاه الكتف، وثلاث عمليات لاستئصال الرحم، وحالتين لاستكشاف البطن لحالات الحمل خارج الرحم الممزق، وإجراء العديد من فحوصات التوليد والأمراض النسائية باستخدام الموجات فوق الصوتية. كما قام المتدربون أيضًا بتولي 41 حالة من الارتعاج أو مقدِّمات الارتعاج الحادة، و25 حالة من أكبر حالات النزف التالي للولادة، و21 حالة إصابة بالصدمة. بالرغم من أن المتدربين ساعدوا كبار الأطباء في البداية فقط، فإن مستواهم قد تقدم في وقت لاحق من الإشراف المباشر إلى الإشراف غير المباشر ومن ثم إلى مستوى الإدارة المستقلة.

**الدروس المستفادة:**

للتعويض عن نقص الأطباء القادرين على تولي حالات التوليد الطارئة الشاملة ورعاية الأطفال الولدان، يمكن للقابلات من ذوي الخبرة تعلم كيفية تقديم الرعاية الصحية الشاملة لحالات التوليد الطارئة وإجراءات القيام بها. يمكن أن يمثل عملهم الجماعي مع الأطباء قيمة عالية وخاصةً في المستشفيات الريفية الواقعة في البلدان الفقيرة في الموارد.

## 摘要

**问题:**

利比里亚医生的匮乏限制了全方位紧急产科和新生儿护理的发展。

**方法:**

在试点项目中，我们对两位高级助产术的助产士进行培训，以团队合作的方法提供住院高级产科护理。此培训为期两年，由利比里亚产科顾问医师在国家专家的协助下展开。

**当地状况:**

此培训在 CB Dunbar 产科医院进行。这个农村医院每年接收约 2000 名接生，其中有很多呈现并发症。2015 年 2 月，利比里亚仅有 117 名医生。

**相关变化:**

在培训的前 18 个月内，受训人员参与了 236 次剖腹产手术、35 次人工抽空流产后滞留产物、25 次人工取胎盘、21 次阴道臀位分娩、14 次真空吸引术助产、4 次破裂子宫修复、4 次肩难产、3 次子宫切除、2 次异位妊娠破裂剖腹手术和多次产科超声检查。 受训人员还处理过 41 次子痫或重度子痫前期、25 次产后出血和 21 次休克。尽管最初受训人员只是帮助高级医师，但是随后从直接指导变成间接指导，然后受训人员可独立处理。

**经验教训:**

如需平衡紧急助产和新生儿护理医师的缺乏情况，我们可对经验丰富的助产士进行培训，使其能够处理高级助产护理和手术。助产士团队与医师的合作在资源匮乏的农村医院尤其宝贵。

## Резюме

**Проблема:**

Из-за недостаточного количества врачей в Либерии возможности оказания комплексной экстренной акушерской и неонатальной помощи ограниченны.

**Подход:**

В рамках экспериментального проекта двух акушеров обучили передовым методикам родовспоможения и бригадному подходу к современному ведению беременности в больничных условиях. Продолжительность обучения составила два года, и обучением руководил либерийский акушер-консультант при поддержке международных специалистов.

**Местные условия:**

Обучение проводилось в роддоме им. Данбара (CB Dunbar Maternity Hospital). В этом медицинском учреждении ежегодно принимают около 2 000 родов, многие из которых характеризуются осложнениями. В феврале 2015 года в Либерии находилось всего лишь 117 врачей.

**Осуществленные перемены:**

В течение первых полутора лет обучения стажеры приняли участие в 236 операциях кесарева сечения, 35 процедурах ручной эвакуации продуктов зачатия, 25 процедурах ручного отделения плаценты, принятии родов при тазовом предлежании в 21 случае, 14 процедурах вакуум-экстракции, а также участвовали в ведении родов, в которых потребовалось лечение разрыва матки (4 случая), и родов при плечевой дистоции (4 случая), 3 процедурах гистерэктомии, 2 операциях лапаратомии для лечения разрыва плодовместилища при внематочной беременности и большом количестве процедур акушерского ультразвукового обследования. Стажеры также проводили лечение эклампсии или преэклампсии тяжелой степени в 41 случае, остановку массивного послеродового кровотечения в 25 случаях и лечение шока в 21 случае. Хотя изначально стажеры лишь оказывали поддержку врачам-специалистам, впоследствии по мере развития их навыков непосредственное руководство было заменено на косвенное и в итоге они проводили лечение самостоятельно.

**Выводы:**

Чтобы компенсировать недостаток врачей, способных оказывать комплексную неотложную акушерскую и неонатальную помощь, можно обучить опытных акушеров передовым принципам родовспоможения и проведению соответствующих процедур. Их участие в бригаде врачей может быть особенно полезным в родильных домах, расположенных в сельской местности стран с ограниченными ресурсами.

## Introduction

In February 2012, Maternal and Childhealth Advocacy International approached the Liberian Ministry of Health and the World Health Organization (WHO), proposing a collaborative partnership to reduce rates of maternal and neonatal mortality in Liberia. One aim was to address the shortage in doctors by training experienced midwives in advanced obstetrics. In October 2012, a formal partnership – including a pilot project training two experienced midwives – was established.

There are at least three reasons for the major shortage of doctors in Liberia. One is the armed conflict that ravaged the country between 1989 and 2003. Another is that more than three quarters of doctors trained in Liberia emigrate to practice elsewhere.^1^ Finally, 184 health workers in Liberia died from Ebola virus disease in the 2013–2016 outbreak.^2^ According to the ministry of health, only 117 doctors were available in the country in February 2015.^2^

In 2012, the United Nations Children’s Fund reported that Liberia had 990 maternal deaths per 100 000 live births, 34 neonatal deaths per 1000 live births and a lifetime risk of maternal death of one in 20.^3^ The corresponding values reported in 2014 were similar: 990 maternal deaths per 100 000 live births, 27 neonatal deaths per 1000 live births and a lifetime risk of maternal death of one in 24.^4^

In September 2013 – after a debate supported by the ministry of health – the Liberian Medical and Dental Council approved the provisional registration of the first two midwives to train as obstetric clinicians. The midwives’ selection for this pilot study was based on their five to six years’ experience in midwifery, the quality of their work in public health facilities and their performance on an internationally accredited three-day training course in emergency obstetric and neonatal care held in Liberia.^5^

## Approach

Apprenticeship-based training in advanced obstetric care was undertaken in CB Dunbar Maternity Hospital, which lies in Bong county – a rural area of almost 9000 km^2^ with a population of about 330 000. This hospital is the county’s main provider of maternity care, caring for approximately 2000 deliveries per year – many seriously complicated by poverty and by delays in transfer from remote villages.

A curriculum was provided by Maternal and Childhealth Advocacy International^6^ and education was led by a Liberian consultant obstetrician with support from two international obstetricians and a professor of paediatrics.

The education materials provided to the trainees and trainers included a practical manual^7^ and pocket book^8^ produced by Maternal and Childhealth Advocacy International, a manual of basic practical skills in obstetrics and gynaecology,^9^ a manual on diagnostic ultrasound^10^ and a textbook on surgical care.^11^ Videos of maternal and neonatal care produced by the Advanced Life Support Group, Global Health Media, Maternal and Childhealth Advocacy International, Medical Aid Films and WHO were also used.

The training began in October 2013 – i.e. a few weeks after preregistration on 3 September 2013. The first three months consisted of a mixture of theory and practice, as outlined in the curriculum.^6^ This foundation training covered knowledge of the anatomy of the female pelvis, basic surgical skills such as suturing, sterilization of instruments, hand washing and the proper use of gowns and gloves, maintenance of the operating theatre, postoperative care, the obstetric use of ultrasound and a basic understanding of obstetric anaesthesia. With the ultrasound, trainees were expected to learn to recognize malpresentations, placenta praevia and other possible problems that may make surgery difficult. At the end of the foundation period, the Liberian and international trainers used an objective structured clinical examination in obstetric anatomy and basic surgical skills to determine the trainees’ progress.

The trainees’ practical skills were increased in the apprenticeship-based training that ran for two years – initially in parallel with classroom-based foundation training. Apprenticeship-based training consisted of a mixture of work experience – when the trainees undertook essential obstetric procedures and treated major complications of pregnancy and delivery – and training in systems of care and team working.

The trainees were given increasing levels of responsibility and involvement over time. At first, the trainees just assisted a senior doctor but, as time passed, they became the primary people undertaking the procedures, albeit always with a senior doctor in the hospital who could give advice or assistance. At this stage, a trainee would often be working with – and supervising – a junior doctor, a peri-operative nurse or at least one other midwife. As time passed, the trainees were given more independence. For example, they were allowed to perform caesarean sections either with someone who had not been trained to do the procedure or with someone who had been trained but played no active role in the surgery. At this stage, a senior doctor was always available to give advice but that doctor could be off-site – e.g. asleep at home – or working elsewhere in the hospital. Records of each procedure involving a trainee were kept in a paper logbook and on a tablet computer.

For quality control, there was continuous clinical assessment of the trainees and examination of the procedural logbook, case-based discussion, supervisor-observed experience and reflective practice forms.

At the end of two years, the trainees were given internships during which they are to continue their apprenticeship-based training for at least one year in Liberian hospitals, supervised by senior doctors. During internships, each trainee will undergo a period of observation – as she undertakes advanced procedures – by Liberian Medical and Dental Council officers, the main Liberian trainer and an international obstetrician. If these observers see evidence of adequate skills and knowledge, and if the trainees pass a final, written examination, trainees will receive a five-year licence to practice as obstetric clinicians in public hospitals chosen by the Liberian Ministry of Health.

Both trainees received their basic hospital salary throughout the project. They also received incentives of 150 United States dollars (US$) per month from Maternal and Childhealth Advocacy International during their apprenticeship, which increased to US$ 300 when they became interns.

## Results

Both trainees successfully completed the objective structured clinical examination and scored high marks in surgical equipment and practice (17/20 and 17/20), opening and closing the abdomen (21/27 and 22/27), uterine anatomy (25/33 and 23/33), bony pelvis anatomy (17/20 and 13/20) and vulval and vaginal anatomy (34/40 and 31/40). Overall scores for the two trainees were 114/140 (81%) and 106/140 (76%).

During their apprenticeship-based training, the two trainees were closely involved with hundreds of advanced obstetric procedures and the management of many serious complications of pregnancy or delivery (Table 1). The trainees participated – often in a leadership role – in the management of all activities within the maternity unit and helped to ensure that the labour and delivery wards and operating theatres were well organized, effective and safe (Box 1). They worked as part of the medical team on shifts of 48–72 hours, supported the senior doctors and helped to train junior doctors. Although there was considerable personal risk, the trainees and their Liberian trainers worked through the outbreak of Ebola virus disease. Support from the trainees – especially at night – helped to provide better sleep patterns for the hospital’s doctors. Although many of the women treated by the trainees were seriously ill, none of them died. The trainees were involved in the successful resuscitation of 73 neonates who did not breathe at birth. At the time of writing, both trainees are awaiting final licencing by the Liberian Medical and Dental Council.

**Table 1:** Actions of the two trainees during the first 18 months of their preregistration training, Liberia, 17 October 2013–31 March 2015

Trainee action^a^	No. undertaken^b^
**Procedure**	
Caesarean section	236
Trainee as assistant	77
Trainee under direct supervision	52
Trainee under indirect supervision	69
Trainee managing procedure independently	38
Manual vacuum aspiration for miscarriage	42
Manual removal of placenta	25
Vaginal breech delivery	21
Vacuum delivery	14
Repair of ruptured uterus^b^	4
Shoulder dystocia	4
Hysterectomy^c^	3
Destructive procedure^c^	2
Laparotomy for ruptured ectopic pregnancy^c^	2
**Management of serious complication**	
Eclampsia or severe pre-eclampsia	41
Postpartum haemorrhage	25
Shock	21

^a^ Training took place at the CB Dunbar Maternity Hospital in Gbanga, Bong county. In this hospital, during the period covered in this table, there were 3017 births and 648 caesarean sections and the two trainees successfully resuscitated 73 neonates, failed to resuscitate 12 neonates, recorded 39 cases of intrauterine fetal death and treated 386 women – none of whom died.

^b^ Some of the patients treated by the trainees required both a procedure and the management of a serious complication.

^c^ Trainee(s) only assisted with this procedure.

Box 1 **Summary of main lessons learnt**

- Experienced midwives can become obstetric clinicians by being taught the skills needed to undertake advanced obstetric procedures such as caesarean sections.
- Obstetric clinicians can work well within a maternity team, assisting doctors and providing improvements in comprehensive emergency obstetric and neonatal care.
- Obstetric clinicians are likely to be particularly valuable in rural hospitals in resource-poor countries where there are few doctors trained in advanced obstetrics.

Rates of maternal and neonatal mortality in Bong county were documented. However, the potential impact of the training programme on these rates was difficult to evaluate because of the outbreak of Ebola virus disease. As the programme continues, its impact on mortality and morbidity should be easier to document.

## Discussion

It appears that, before this pilot project, there was no formal training for midwives to undertake advanced surgical obstetric care.^12^ The arguments as to why such training is needed in many rural areas of sub-Saharan Africa where there are few doctors and under special circumstances have been summarized elsewhere.^12^

A year of negotiations was necessary before the Liberian Medical and Dental Council were willing – provisionally – to register the first two trainees. The delay reflected doubts from some doctors about the likely benefits. A senior doctor who began working with the two trainees halfway through their training was initially worried about whether midwives could ever safely undertake procedures such as caesarean sections. After a few weeks working alongside the two trainees, however, this doctor became convinced that this approach represents a necessary and effective way forward for rural hospitals in Liberia.

The Liberian Ministry of Health has now agreed to increase the number of obstetric clinicians in Liberia, via a second round of training. Another seven experienced midwives started training in October 2015 – together with two physician assistants with extensive experience in midwifery. Four of the new trainees come from a hospital in a remote county of Liberia where there is only one doctor undertaking advanced obstetric surgery. Once trained, some of these four trainees will return to work long-term in this hospital.
